# Supplementary material for: Plant-Derived Oleanolic Acid (OA) Ameliorates Risk Factors of Cardiovascular Diseases in a Diet-Induced Pre-Diabetic Rat Model: Effects on Selected Cardiovascular Risk Factors
Source: Molecules. 2019 Jan 18;24(2):340. doi: 10.3390/molecules24020340 (PMC6359497; doi:10.3390/molecules24020340)
Supplement: Supplementary file 1 [file molecules-24-00340-s001.pdf]

Supplementary Materials

Plant-Derived Oleanolic Acid (OA) Ameliorates Risk Factors of Cardiovascular Diseases in a Diet-Induced Pre-Diabetic Rat Model: Effects on Selected Cardiovascular Risk Factors

Mlindeli Gamede, Lindokuhle Mabuza, Phikelelani Ngubane and Andile Khathi *

Department of Human Physiology, School of Laboratory Medicine and Medical Sciences, College of Health Sciences, University of KwaZulu-Natal, X54001 Durban, South Africa; 213571877@stu.ukzn.ac.za (M.G.); 211509843@stu.ukzn.ac.za (L.M.); Ngubanep1@ukzn.ac.za (P.N.)

* Correspondence: khathia@ukzn.ac.za; Tel.: +27-0312-607-585

Table S1: Composition of the high fat high carbohydrate (HFHC) diet

Ingredient	Incl(%)	Mix(kg)
Maize	38.98	390.000
Palm Oil	20.99	210.000
Soya Full Fat	14.99	150.000
Wheat Gluten	6.50	65.000
Flour	6.00	60.000
Monodex	5.00	50.000
Sugar - White	5.00	50.000
Limestone	1.00	10.000
Dicalcium Phosphate	0.50	5.000
Vitamin Premix	0.35	3.500
Salt - Fine	0.30	3.000
Amino Acid - DL Methionine	0.30	3.000
Mineral Premix	0.10	1.000
	**100.01**	**1000.50**

Table S2: Nutritional value of the high fat high carbohydrate (HFHC) diet

Nutrient	Units	Actual
Dry Matter	g/kg	919.93
Metabolizable Energy	MJ/kg	15.86
Crude Protein	g/kg	151.27
AShreonine	g/kg	4.51
ASIsoluecine	g/kg	5.24
ASLysine	g/kg	6.54
ASMethionine	g/kg	4.86
ASryptophan	g/kg	1.30
ASstidine	g/kg	3.30
ASTSAA	g/kg	6.79
ASValine	g/kg	5.80
Fat	g/kg	250.46
Carbohydrate	g/kg	427.29
Fibre	g/kg	22.08
Ash	g/kg	26.31
Avl Phosphorus	g/kg	1.66
Calcium	g/kg	5.47
Total Phosphorus	g/kg	3.60

Table S3: Composition of fats, proteins and carbohydrates of the normal diet (ND) and high fat high carbohydrate diet (HFHC)

	Fats (%)	Proteins (%)	Carbohydrates (%)
**ND**	15	30	55
**HFHC**	25	15	60
